# Respect for Autonomy and Dementia Care in Nursing Homes: Revising Beauchamp and Childress’s Account of Autonomous Decision-Making

**DOI:** 10.1007/s11673-022-10195-7

**Published:** 2022-06-24

**Authors:** Hojjat Soofi

**Affiliations:** 1 Department of Clinical Medicine, Macquarie University, Level 1, 75 Talavera Rd, Macquarie Park, NSW 2113 Australia; 2 Department of Philosophy, Macquarie Univesity, Levels 6, 25B Wally’s Walk, Macquarie Park, NSW 2109 Australia

**Keywords:** Respect for autonomy, Informed consent, Dementia, Nursing homes

## Abstract

Specifying the moral demands of respect for the autonomy of people with dementia (PWD) in nursing homes (NHs) remains a challenging conceptual task. These challenges arise primarily because received notions of autonomous decision-making and informed consent do not straightforwardly apply to PWD in NHs. In this paper, I investigate whether, and to what extent, the influential account of autonomous decision-making and informed consent proposed by Beauchamp and Childress has applicability and relevance to PWD in NHs. Despite its otherwise practical orientation and suitability for acute care settings, I identify three problems with this account when applied to PWD in NHs. These problems include (1) intentionality as an all-or-nothing condition of autonomous decision-making, (2) construing consent as one-off authorization, and (3) unresolved ambiguities around the primacy of precedent autonomy over best interest considerations. To address these problems, I propose and defend a number of revisions to Beauchamp and Childress’s account. First, I suggest that we consider intentionality as a non-binary criterion of autonomous decision-making. Second, I argue for a model of process consent to overcome the moral inadequacy of construing consent as one-off authorization in NHs. And, to overcome the third problem, I suggest accounting for both precedent and extant autonomy of PWD, considering mandates of precedent autonomy not as prescriptive but as informative, and drawing a less rigid distinction between autonomy considerations and best interest judgements. I conclude that this revised version of Beauchamp and Childress’s account fares better than the original version in capturing relevant autonomy considerations to care for PWD in NHs.

## Introduction

Conceptions of personal autonomy in biomedical ethics differ in two main ways from those that have appeared in the wider philosophical literature.

First, theories of personal autonomy in biomedical ethics tend not to commit to overly demanding ideals of autonomy. The key idea here is that overly demanding standards of autonomy, for example, of the sort that requires higher-order reflection (Frankfurt 1971), do not suit the practical purpose of invoking autonomy considerations in biomedical ethics, which is to counter the threat of deep-rooted paternalism in clinical encounters.

Second, accounts of personal autonomy in biomedical ethics concentrate on *local* autonomy (being autonomous with respect to particular decisions) instead of *global* autonomy (being an autonomous person) (Mackenzie 2015). The suggested reason for adopting this approach is that many individuals who are generally capable of self-governance (i.e., autonomous persons) might “fail to govern themselves in particular choices” (Beauchamp and Childress 2013, 104).

As such, theories of autonomy in biomedical ethics seem to be better suited to assessing particular decisions in clinical contexts than (most) conceptions of autonomy in the wider philosophical literature. Despite this, there remain a number of challenges when assessing and accounting for the autonomy of groups of patients such as people with dementia (PWD) in nursing homes (NHs). As Agich notes, received notions of autonomy and informed consent in biomedical ethics are developed primarily to account for the autonomy of patients in acute care settings and in situations that present “conflictual, dramatic, and discrete characteristics” (2003, 10). Agich suspects that this primary orientation to acute care settings may have rendered received notion of autonomy and informed consent in biomedical ethics unable to be adequately “interstitial to the typical everyday reality of long-term care” (2003, 10). Similarly, Pullman (1999) raises concerns about the applicability and relevance of received notions of autonomy and informed consent in biomedical ethics to long-term care. Pullman even goes further. He is sceptical of attempts to revise received notions of autonomy in biomedical ethics to improve their practical relevance to long-term care. Pullman holds that many of such revisionary attempts “either fail the test of practical relevance when applied in the context of long-term care, or, they become conceptually obtuse” (1999, 30).

Against this background, in this paper, I set out to investigate whether, and to what extent, the account of autonomous decision-making offered by Beauchamp and Childress (2013) captures and accounts for relevant autonomy consideration to care for PWD in NHs. I focus on this account as it has gained considerable traction and has even been considered to be the “clearly dominant account of autonomy” (Kukla 2005, 35) or “the default conception” of autonomy (Dive and Newson 2018, 172) in biomedical ethics.

The paper is structured as follows. Section 1 gives an overview of Beauchamp and Childress’s account of autonomous decision-making/informed consent. Section 2 discusses how PWD in NHs pose a number of problems for the account of autonomy/informed consent developed by Beauchamp and Childress. In doing so, I draw on Agich’s critique of received notions of autonomy and informed consent in biomedical ethics, but I expand and move beyond his critique. Then, in Section 3, I discuss and defend several revisions to Beauchamp and Childress’s account. The last section concludes and summarizes the discussions in this paper.

## Beauchamp and Childress’s Account of Autonomous Decision-Making

Beauchamp and Childress lay down three non-ideal conditions for an act to be considered autonomous: *intentionality*, *understanding,* and *voluntariness* (or *non-control*).

According to Beauchamp and Childress, for an act to meet the criterion of intentionality, it needs to correspond to the actor’s plan. This contrasts with accidental actions in which there is no correspondence between the action and the actor’s plan. The intuition supporting the claim that accidental actions are not autonomous is that to exercise autonomy, an actor should have some control over the action in the form of prior planning or goal setting.

The second condition of autonomous acts, namely understanding, requires ensuring that an actor comprehends the meaning and consequences of her action. The criterion of understanding is a non-ideal condition: meeting the criterion of understanding does not demand full understanding. It also does not require the total absence of constraining influences on understanding such as the presence of disease-related discomfort or anxiety, which can be a bar to understanding. The presence of a sufficient level of comprehension suffices for an act to meet the criterion of understanding.

The third condition is voluntariness or absence of controlling influences. It denotes freedom from either external or internal sources of control “that rob the person of self-directedness” (Beauchamp and Childress 2013, 104). External sources of control refer to controlling influences exerted by one person over another. Internal sources of control, on the other hand, refer to constraining influences such as mental illness, which may hinder patients’ control over what they do.

On Beauchamp and Childress’s account, the criteria of understanding and voluntariness are matters of degree. Patients’ understanding can be on a continuum from complete understanding to the total absence of understanding (e.g., a patient in a coma). Likewise, there might be a range of freedom from being under no control to being completely constrained (e.g., a patient with an extreme form of psychosis). By contrast, Beauchamp and Childress hold that the criterion of intentionality cannot be a matter of degree. That is, according to them, “acts are either intentional or nonintentional” (2013, 105).

According to Beauchamp and Childress, patients’ decisions^1^ that do not meet the three conditions of autonomous decision-making are considered either “doubtfully autonomous” or “nonautonomous” (2013, 226).^2^ This, however, does not mean that in caring for these patients, there are no autonomy-related ethical considerations.

Some patients, whose current decisions do not satisfy the three criteria of autonomous decision-making, might have been competent in the past to make autonomous choices. For instance, people with severe dementia might not be able now to make autonomous decisions, but many of their past decisions can be considered autonomous prior to the onset of cognitive decline. For these patients, Beauchamp and Childress hold, clinical decision-making should focus on their precedent autonomy.

The general idea motivating appeals to precedent autonomy is that the values and preferences of now-incompetent but formerly competent patients should be respected. As such, appeals to precedent autonomy aim to extend the ethical obligations of respect for autonomy when patients lose their competence for autonomous decision-making.

While, according to Beauchamp and Childress, now-incompetent but formerly competent patients should generally be treated according to their precedent autonomy, in some cases, patients’ former preferences cannot be identified. For instance, a surrogate decision-maker might not be familiar enough with the patient to determine what she would have decided if competent, or there might be no formal advance directive stating the patient’s prior preferences. In such cases, Beauchamp and Childress concede that appeals to precedent autonomy lose their moral binding force.

Instead, Beauchamp and Childress introduce another approach to surrogate decision-making. According to the best interest approach, when appeals to precedent autonomy are not practically feasible, surrogate decision-makers should make the choice that results in “the highest probable net benefit among the available options” (Beauchamp and Childress 2013, 228).

The aforementioned account of prioritizing precedent autonomy over the best interest approach comes with an important caveat. In rare cases, Beauchamp and Childress hold, even if we know patients’ former preferences, we might justifiably override them and adopt the best interest approach.

In arguing so, Beauchamp and Childress refer to the widely discussed case of Margo, a person with dementia of Alzheimer’s type whose advance directive states that she does not want any life-sustaining treatment if she develops a life-threatening illness. Margo now seems happy and takes pleasure in everyday activities.

Given her current situation, scholars have posed the question as to whether it is justified not to treat Margo with antibiotics if she develops severe, life-threatening but treatable pneumonia.

Beauchamp and Childress, *pace* scholars such as Dworkin (1993), hold that the case of Margo is an exception to their general view on prioritizing precedent autonomy over best interest considerations. This leads them to conclude that while appeals to precedent autonomy generally come before best interest considerations, in rare cases (e.g., in the case of Margo), we can justifiably override patients’ former preferences in favour of their current experiential interests.

The above was a brief overview of Beauchamp and Childress’s account of autonomous decision-making in healthcare contexts.

According to Beauchamp and Childress, their account of autonomous decision-making provides ground for defining informed consent requirements, which are the most salient autonomy-related ethical considerations in clinical contexts. By appealing to their three-condition theory of autonomous choice, Beauchamp and Childress argue that rigorous informed consent procedures require that a patient with “substantial understanding and in the absence of substantial control by others, intentionally authorizes a professional to do something quite specific” (2013, 122).^3^

## The problems of Beauchamp and Childress’s account

In this section, I identify and discuss three issues with Beauchamp and Childress’s account when applied to PWD in NHs.

### Intentionality as an All-or-Nothing Condition of Autonomous Decision-Making

Beauchamp and Childress define the criterion of intentionality in terms of exercising the ability to plan. Their account presumes that agents generally have the ability to plan and if agents perform actions that we have not planned or desired to do, these actions count as unintentional. In other words, as far as intentionality is concerned, there are only two possibilities: an agent plans to do the action she performs, or she does not plan to do so.

This dichotomous conceptualization of intentionality raises questions regarding which decisions made by PWD can be considered autonomous.

There is neuropsychological evidence indicating that dementia negatively affects individuals’ planning abilities (Passini et al. 1995; Rainville et al. 2002; Satler, Guimarães, and Tomaz 2017). The ability to plan is a type of executive function (Rainville et al. 2002). People with diminished executive function experience difficulties in planning and goal-setting, particularly when routine behaviours are not sufficient to deal with new problems (Johns et al. 2009; Passini et al. 1995). Deficits in executive function are part of the symptomology of different types of dementia including dementia of Alzheimer’s type, frontotemporal dementia, and Lewy Body dementia (Johns et al. 2009; Rainville et al. 2002).

Given this clinical picture, the ability to plan in PWD can be graded along a continuum from full intentionality (i.e., as intentional as generally competent, neurotypical persons) to almost non-intentional (i.e., near-total loss of ability to plan). Beauchamp and Childress’s account is fairly clear that actions taken by people with significant loss of ability to plan do not meet the criterion of intentionality, and as such, cannot be considered autonomous. People with very advanced stages of dementia might fall into this category.

But what about people with mild to moderate dementia whose ability to plan is compromised? These individuals, unlike people at very advanced stages of dementia, retain some (and at times, even considerable) control over what they do via former planning and goal-setting.

This is consistent with findings of empirical research investigating deficits in the ability to plan in people with mild to moderate dementia by using assessment tools such as the Tower of London test (Rainville et al. 2002). This research shows that people with mild to moderate dementia have the same success rate in solving the simplest planning test as the control group. But, as the complexity of the tests increases, both the control group and the participants with mild to moderate dementia show decreased levels of performance. The participants with mild to moderate dementia, nonetheless, were less successful than the control group in solving the more complex tests. This suggests that the ability to plan is diminished, yet still present to some extent, in people with mild to moderate dementia.

Similarly, another study by Passini et al. (1995) investigated the ability to plan in terms of a complicated wayfinding task in a group of elderly participants with mild to moderate dementia of Alzheimer’s type. The researchers found that although none of the participants managed to arrive at the destination, the participants were able to create sub-plans in familiar situations and when the necessary information was easily available to them. Based on these findings, Passini et al. suggest that some basic ability to plan seems to be extant in people with mild to moderate dementia, particularly when there are clear informational clues in the surrounding environment.

Can we then consider acts taken by people with mild to moderate dementia with diminished ability to plan as intentional?

Clearly, these acts do not match the general description of intentional acts proposed by Beauchamp and Childress, in which a neurotypical person with uncompromised ability to plan intends to perform a particular course of action. Nonetheless, acts taken by people with mild to moderate dementia with diminished ability to plan do not fit the corresponding description of non-intentional (accidental) actions as well. Such acts, therefore, do not seem to fit into either of the two categories of intentional or non-intentional (accidental) acts as construed by Beauchamp and Childress.

Are these acts autonomous? Beauchamp and Childress’s dichotomous account gives us little direction in answering this question. This creates a problem for Beauchamp and Childress’s account when applied to PWD. I call this *the problem of intentionality as an all-or-nothing condition of autonomous decision-making*.

### Consent as One-Off Authorization

There is a second problem with Beauchamp and Childress’s account when applied to PWD in NHs. The problem arises from Beauchamp and Childress’s assumption as to what are relevant loci of exercising autonomy. Given that Beauchamp and Childress mainly focus on autonomous decision-making in acute care settings, their account assumes that relevant loci of exercising autonomy in healthcare contexts are discrete, specific, and temporally limited decisions.

As I mentioned in the introduction, and in agreement with Agich (2003), I think these assumptions do not straightforwardly apply to decision-making in long-term care settings. While patients in acute care settings receive discrete, short-term interventions, in NHs there are interrelated and temporally extended episodes of care.

Management of behavioural and psychological symptoms of dementia (BPSD) in NHs is a prime example. This type of care requires regular assessments, ongoing monitoring of how residents with BPSD respond to treatments, and adjusting treatment plans according to their responses (Kales et al. 2019). These steps constitute an ongoing cycle of care, which requires frequent communication with residents with BPSD in NHs. Each of these communications might be an opportunity to involve the residents in the process of decision-making, solicit their preference for the care they receive, and possibly revise or reverse a previous decision.

The potential loci of exercising autonomy in NHs then seem to be different from acute care settings. Not only are the loci of exercising autonomy in NHs manifold, but they are also implicationally related to each other. This means that residents in NHs cannot make temporally limited decisions in isolation from each other. The assumption that loci of exercising autonomy are discrete and time-limited decisions, although reasonable in the context of acute care settings, does not jibe with residents’ decision-making about the care they receive in NHs. As such, it seems ethically inadequate to obtain a single authorization from PWD in NHs. I call this *the problem of consent as one-off authorization*.

### Ambiguity Concerning the Primacy of Precedent Autonomy Over Best Interest

The third issue with Beauchamp and Childress’s account arises from ambiguities about when patients’ present best interest considerations can justifiably override mandates of precedent autonomy. In section 1, I discussed how the case of Margo, according to Beauchamp and Childress, serves as an example of exceptional circumstances in which best interest considerations trump the patients’ former preferences.

But why is the case of Margo an exception to the general rule that precedent autonomy comes before best interest considerations? Beauchamp and Childress do not address this question in detail. In what follows, I offer two possible explanations and show that even the more appealing explanation leaves some questions unanswered.

The first possible explanation is that the case of Margo is an exception because of the significance of the patient’s current experiential interests. This explanation, however, needs to qualify what constitutes significant experiential interests. Margo does not seem to be in a different situation from many PWD in NHs, who despite significant cognitive decline, seem to have an interest in and enjoy everyday activities. Accepting this explanation then broadens the scope of exceptions we make to the general rule such that the general rule (prioritizing precedent autonomy over best interest judgements) would not apply to most cases of PWD in NHs. This defies the purpose of establishing a general rule in the first place.

Another possible explanation is that the case of Margo can be considered an exception not because of the importance of her current experiential interests but because of the *significance of the conflict* between what respecting precedent autonomy requires and the action dictated by our best interest judgements. According to this explanation, we need to find what actions precedent autonomy requires in any given case. Then, we have to clarify whether there is any conflict between actions mandated by precedent autonomy and our best interest judgements. Beyond a certain threshold, significant conflicts provide ground for waiving the general rule, and we can justifiably act on best interest judgements.

This latter explanation might seem more straightforward and, thus, appealing. But some questions remain unanswered.

Why should we limit the scope of best interest considerations to cases in which they come into *significant* conflict with precedent autonomy? What counts as significant conflict here? What about cases when best interest considerations are at some, although not considerable, variance with requirements of precedent autonomy?

Addressing these questions requires us not only to clarify why the case of Margo is an exception but, more broadly, problematizes the very idea that the requirements of precedent autonomy should be generally prioritized over best interest judgements. This raises another problem for Beauchamp and Childress’s account when applied to PWD in NHs. I call this *the problem of prioritizing precedent autonomy over best interest considerations*.

## Addressing the Problems of Beauchamp and Childress’s Account

This section attempts to address the three problems that I discussed above. I suggest and argue for a number of revisions to Beauchamp and Childress’s account to overcome these problems.

### The Problem of Intentionality as an All-Or-Nothing Criterion of Autonomous Decision-Making

Below, I show that depending on how we interpret Beauchamp and Childress’s account, there might be at least two possible solutions to the problem of intentionality as an all-or-nothing criterion of autonomous decision-making.

Applying a strict interpretation of Beauchamp and Childress’s account leads us to consider actions taken by patients with any diminution in intentionality as being non-autonomous. In other words, according to this interpretation, all decisions made by people with diminished ability to plan are, in fact, unintentional and, as such, non-autonomous. The consequence then would be to consider all PWD with any diminution in intentionality (i.e., the ability to plan) as incompetent to consent to care they receive in NHs. This is a straightforward approach to solve the problem of intentionality as an all-or-nothing criterion of autonomous decision-making. This approach, however, suffers from a significant problem.

It does not make good intuitive sense to equate actions taken by people with diminished ability to plan with unintentional actions. Unintentional (or accidental) actions occur when a person, who is generally capable of prior planning and goal-setting, acts in a way that does not accord with what she had in mind to do before taking action. This incongruity between prior planning and the act in question is not the case with all actions taken by people with diminished ability to plan.

People with diminished, yet still extant, ability to plan can act in a way that, to some extent, aligns with their prior ideas of what activities they want to perform. For instance, an individual at early stages of dementia of Alzheimer’s type might experience difficulties in planning her daily schedule. Despite such difficulties, she may well perform most of the activities included in her schedule. This means that from the fact that an actor has diminished ability to plan, it does not follow that there is a complete mismatch between what she plans to do and the actions she actually takes. Therefore, the strict interpretation of Beauchamp and Childress’s account seems intuitively implausible.

There is, nonetheless, another possible solution to resolve the said problem. Similar to the criteria of understanding and voluntariness, we can revise Beauchamp and Childress’s account to consider intentionality as a non-binary criterion.

While the strict interpretation of Beauchamp and Childress’s account unduly disregards all acts taken by people with diminished ability to plan as non-autonomous, this modified interpretation allows more flexibility in assessing the degree to which decision-making by PWD is autonomous. In certain cases, some acts taken by people with diminished intentionality can meet the modified non-binary criterion of substantial intentionality and can be considered autonomous.

Consider, for instance, Mr T, a person at an early stage of Alzheimer’s disease who has been recently admitted to a NH. Prior to the admission, Mr T was given risperidone (an antipsychotic medication) by his family members in his home. After examining Mr T’s clinical profile, the physician on duty decides to gradually taper off the medication over a one-month period and replace it with another medication. During this period, however, the antipsychotic medication needs to be still administered to Mr T although with gradually decreasing doses. The staff in the NH are instructed to obtain informed consent from Mr T, who experiences some deficits in his ability to plan such as not foreseeing the consequences of actions he takes to the full extent that a neurotypical person would foresee. Nonetheless, with simplified information in place, Mr T is still able to set fairly clear goals for his treatment plan as a whole and the temporary role that the antipsychotic therapy plays in the plan. Mr T gives consent to the use of antipsychotic treatment with the deprescribing plan in place.

In this example, Mr T’s ability to plan is diminished. The strict interpretation of Beauchamp and Childress’s account would judge that Mr T is incompetent to consent to the antipsychotic treatment because of the diminution in his intentionality. This, nevertheless, does not seem to be sound judgement. Mr T still has substantial intentionality. The proposed non-binary notion of intentionality, however, allows us to judge the decision made by Mr T to receive the antipsychotic medication as autonomous: Mr T's decision is sufficiently intentional to be considered autonomous.

But what about those actions taken by people with diminished ability to plan that are not sufficiently intentional to be considered autonomous? To answer this question, I suggest that we make a further revision to Beauchamp and Childress’s account.

**Fig. 1:**
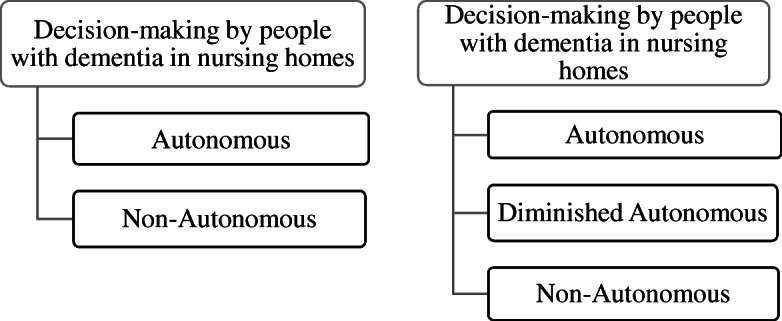
Categories of decision-making as per the original version of Beauchamp and Childress’s account (on the left) and the revised account (on the right)

I suggest that instead of having two categories for classifying decision-making by PWD (i.e., autonomous/non-autonomous), we need three categories: *autonomous*, *diminished autonomous*, and *non-autonomous* (Figure 1).

The above suggestion implies that we need to construe autonomous decision-making by PWD as a matter of degree. In the following two sections, I apply this revised classification of decision-making by PWD in NHs.

Before going on to the next section, I wish to address one possible objection to the revised version of Beauchamp and Childress’s account as sketched above.

The objection is that it is unclear what should count as sufficient intentionality. Although I acknowledge that further clarifications are necessary, we can think of the same objections regarding the criteria of understanding and voluntariness. Beauchamp and Childress themselves note that the line between the adequate and inadequate degree of understanding and voluntariness might appear arbitrary (2013, 105). They, however, emphasize that the thresholds need to be set taking into consideration “specific objectives” of the acts in question (2013, 105).

### The Problem of Consent as One-Off Authorization

This section turns to the second problem, namely, the problem of consent as one-off authorization. To that end, I introduce an alternative model of consent which, while compatible with Beauchamp and Childress’s account, is better able to overcome the objection raised in section 2.2.

Before that, one clarification is in order. The discussions in this section relate to those cases in which PWD in NHs can make decisions with sufficient intentionality, understanding, and voluntariness. My working assumption is that to respect the autonomy of these individuals, who may constitute a small minority of residents in NHs, we must seek their informed consent but use a modified approach. Note, however, that I am not implying that diminished autonomous decision-making does not command any moral respect. I offer an alternative way of respecting diminished autonomous decision-making in the following section (3.3).

The model of consent that I aim to develop here builds on what has been called process consent in the context of qualitative research (Houghton et al. 2010; Munhall 1988, 1991; Raudonis 1992). Qualitative research such as ethnographic studies requires observations of research participants over an extended period of time. Further, researchers who conduct qualitative research may conduct multiple interviews with the same research participants. In many cases, during the course of conducting such studies, researchers might change the focus of research or may discover unanticipated findings (Munhall 1991). In such situations, it is suggested that the researchers should renegotiate the aims/methods of the study through ongoing consensual engagement with research participants (Munhall 1991). Process consent denotes such ongoing arrangements to, among other things, inform research participants about the changes to the focus of the study and reach an agreement with them before conducting further observation or interviews (Munhall 1991).

Usher and Arthur (1998) argue that the notion of process consent is also useful in the context of mental health nursing, in which changes to patients’ treatment plans are common. In particular, they consider process consent as a counter to the common assumption in psychiatric practice that seeking informed consent is a one-off event and may “last forever” (696). Process consent, according to Usher and Arthur, requires ongoing communication with patients and seeking their consent in every stage of the treatment plans.

In line with the foregoing, the idea of process consent is also useful to develop a solution to the problem of consent as one-off authorization. I propose two steps that need to be taken to adopt a model of process consent in NHs.

First, consent needs to be sought on an ongoing basis. This ensures that, for instance, residents do not consent to the use of psychotropic medications at the time of admission to NHs and continue receiving the medications for long periods of time. Instead of focusing on one specific moment, ongoing consensual engagement with residents in NHs distributes loci of exercising autonomy throughout their treatment plans. Second, it might be more helpful to consider consent not as stand-alone moments of autonomous decision-making but as parts of wider decisions that residents make in NHs to determine the overall shape of care they receive.

Is this alternative model of consent compatible with Beauchamp and Childress’s account?

It seems that it is, although now their account needs further revisions. The model of process consent that I sketched above is neither a one-off nor a decision-specific approach to seeking consent from residents in NHs. In this way, the model of process consent differs from Beauchamp and Childress’s account, which in its original version focuses on specific, punctate decisions.

But we have to bear in mind why Beauchamp and Childress take up a different conception of personal autonomy than those discussed in the wider philosophical literature. Beauchamp and Childress rely on a less stringent conception of autonomy because they want to optimize their account for assessing autonomous decision-making in healthcare contexts.

Likewise, I suggest adopting the process consent model because it is better suited for seeking consent from PWD in NHs. I simply extended Beauchamp and Childress’s reasoning (i.e., the need to have a better-optimized account) to the context of decision-making by residents with dementia in NHs.

Is my suggested model of process consent in NHs different from Usher and Arthur’s notion of process consent? Yes, because Usher and Arthur propose seeking informed consent every time medications are administered to patients. I think this is a very onerous task. Instead of seeking consent, for instance, every time antipsychotic medications are administered to PWD in NHs, I think, it might be more practical to ensure regular consensual engagement with patients when major changes to treatment plan (e.g., significant increase of dosage or implementing a deprescribing plan) take place.

### The Problem of Prioritizing Precedent Autonomy Over Best Interests

I now aim to address the third problem, namely, the problem of prioritizing precedent autonomy over best interest considerations. Most of the discussions in this section focus on diminished autonomous decision-making (figure 1). Towards the end of the section, nonetheless, I discuss the case of PWD whose decision can be properly considered *non-autonomous* (figure 1). I note that such cases remain more challenging, but I suggest possible ways to ease the task of balancing mandates of precedent autonomy against best interest considerations.

I will take three steps to address the problem of prioritizing precedent autonomy over best interest considerations. I argue for (1) accounting for both precedent and extant autonomy of PWD, (2) considering mandates of precedent autonomy not as prescriptive but as informative, and (3) the need to draw a less rigid distinction between autonomy considerations and best interest judgements.

My first step is to challenge the assumption that the only relevant autonomy considerations with respect to people with diminished capacity for autonomous decision-making are what their precedent autonomy dictates. Note that, in these cases, Beauchamp and Childress’s assumption that precedent autonomy should generally be prioritized over best interest considerations does not mean that we must decide between two different autonomy standards. On their account, the only relevant autonomy standard is what patients’ precedent autonomy mandates. The best interest considerations are of a different kind. Best interest considerations, according to Beauchamp and Childress, take into account patients’ welfare and invoke the obligations of beneficence.

Beauchamp and Childress rightly assert that the requirements of precedent autonomy might be the only autonomy consideration in some cases. I contend, however, that this may not be the case with PWD whose ability to make autonomous decision-making is diminished.^4^

As Shiffrin (2004) avers, the case of PWD with diminished ability to make autonomous decisions is analogous to those cases in which we seem to be required to show moral respect to the preferences of individuals who have never been, are not, and will not be competent to make fully autonomous decisions. Shiffrin gives the example of terminally ill children. I assume that most of us would agree that, despite the fact that these children are not and will not be able to make autonomous decisions, we should respect their preferences.

What is the reason for this? The generic answer to questions of this type is that respecting children’s preferences and decisions is helpful to them to develop their abilities to become fully autonomous in the future (Diekema 2003). This answer, however, does not apply to terminally ill children. Terminally ill children do not reach adulthood, and therefore their ability to make autonomous decisions will never develop to the fullest.

I agree with Shiffrin that we seem to have another reason for taking seriously the preferences of terminally ill children. The reason is that it is valuable for one to exert some control over one’s life even if one is not capable of making autonomous choices in most or all aspects of one’s life. If this is correct, then, we seem to have the same reason to take seriously contemporaneous expressions of autonomy in PWD whose ability to make (fully) autonomous decisions is diminished.^5^

One might, however, reasonably hold that there is a morally relevant difference between PWD and terminally ill children. The former group of individuals, unlike the latter, have been competent in the past to make autonomous decisions. This difference is clearly of significance. But it does not undermine the reason not to take seriously current preferences of PWD who show diminished ability to make autonomous choices. Rather, it implies that, in order to respect their autonomy, we have to take into account *both* mandates of their precedent autonomy and their diminished, yet extant, autonomy.

Now one might raise the question as to how it is possible to morally respect diminished autonomous decision-making.

One possible answer to this question could be to extend the requirements of assent from dementia research to dementia care. In the context of dementia research, it is a widely adopted institutional requirement to obtain assent from participants with dementia who lack the capacity to consent (Black et al. 2010; Slaughter et al. 2007). The underlying assumption behind obtaining assent from research participant with dementia is that it is imperative to respect “individuals’ remaining autonomy” and to provide “the opportunity for cognitively impaired adults to be involved, to the extent possible, in the decision-making process” (Black et al. 2010, 4).

As Dubljević (2020), Coverdale et al. (2006), and Molinari et al. (2004, 2006) argue, the notion of assent can do similar moral work in the context of dementia care. Obtaining assent seems to be a practically viable option to operationalize respect for the extant, although diminished, autonomy of PWD in NHs. To seek assent from PWD, healthcare professionals in NHs need to identify any relevant preferences that residents might have regarding the care they receive. Further, it also requires engaging the residents in decision-making about the care they receive commensurate with their extant decision-making capacities.

Conversely, one example of not giving due respect to the extant autonomy of PWD in NHs with diminished capacity for autonomous decision-making is the practice of covert administration of psychotropic medications such as antipsychotic medications.^6^ This practice involves hiding the medications in the residents’ food or beverages (Kirkevold and Engedal 2005; Treloar, Beats, and Philpot 2000). Although many of these individuals are not able to consent to the use of the medication, the practice is still likely to be autonomy-undermining. This is because notwithstanding the fact that many of them might not understand the specific effect of the tablet in question, they may understand that their care involves the administration of some medications. The practice, then, is contrary to the requirements of assent as I discussed above, given that caregivers do not make any effort to communicate to the individuals the fact that they receive medications and not just food or beverages.

This was my first step towards addressing the third problem of Beauchamp and Childress’s account. I argued that to show due moral respect to the autonomy of PWD in NHs with diminished capacity for autonomous decision-making, we need to account for both their prior wishes and their contemporaneous expressions of autonomy.

The second step that I want to take is to introduce a new approach to accommodating mandates of precedent autonomy of PWD. While Beauchamp and Childress consider mandates of precedent autonomy as *prescriptive*, I will argue that it might be more helpful to consider the mandates as *informative*. In doing so, I draw on a hermeneutic construal of precedent autonomy that has been offered by Widdershoven and Berghmans (2001).

Patients’ precedent autonomy plays a prescriptive role in Beauchamp and Childress’s account. On their account, in the form of advance directives, precedent autonomy provides instructions for healthcare professionals to follow. Likewise, in the form of substituted judgement, a proxy decision-maker constructs the will of a now-incompetent but formerly competent patient and thereby identifies hypothetical instructions to be followed. This prescriptive approach falls within what Widdershoven and Berghmans characterize as the standard bioethical thinking about precedent autonomy.

Widdershoven and Berghmans charge the conventional bioethical thinking about precedent autonomy with not paying heed to the problems of interpretation. Simply put, they argue that putting the mandates of precedent autonomy (particularly in the form of advance directives) into practice requires interpretation and specification of general statements.

Widdershoven and Berghmans argue that adopting the prescriptive approach does not “unambiguously tell us what to do” (2001, 184) and leaves unanswered many “new questions for those who have to make decisions about treatment and care for incompetent patients” (2001, 182). This is consistent with previous empirical research documenting the ambiguities and problems associated with implementing patients’ advance directives (or living wills) and with making substituted judgements in actual clinical settings (Ditto et al. 2001; Li et al. 2007).

Instead, Widdershoven and Berghmans adopt a hermeneutic approach to the requirements of precedent autonomy in the context of dementia care. According to this approach, determining how advance directives create obligations in practice requires considering advance directives as texts, the meaning of which needs to be clarified through further interpretations. This means that advance directives do not prescribe specific courses of action. According to the hermeneutic approach, advance directives are *tools* to inform the deliberation of dementia care providers in NHs to find practical implications of residents’ former preferences.

One advantage of this hermeneutic understanding of the requirements of precedent autonomy is that it can link residents’ precedent autonomy with their contemporaneous expressions of autonomy. As Widdershoven and Berghmans note, many PWD with diminished capacity for autonomous decision-making can still provide input into our deliberations to make meaning of their past preferences and wishes. Their input might not always be coherent or consistent, but they seem to be potential sources of knowledge to better understand the individuals’ long-held values or beliefs.

To sum up the discussions above, I first argued that our autonomy standard for PWD who no longer have substantial ability to make autonomous choices should be defined based on their precedent autonomy and their current expressions of autonomy. Next, by adopting a hermeneutic perspective, I argued that instead of considering the requirements of precedent autonomy as prescriptive, it is more promising to see them as informative. This alternative approach makes our autonomy standard responsive to the requirements of precedent autonomy while making it possible for PWD to express their current preferences, which also guides us, to some extent, in interpreting their past wishes.

And now, I intend to take my third and last step towards developing a solution to the third problem of Beauchamp and Childress’s account. In what follows, I argue that the dichotomy that the original version of Beauchamp and Childress’s account draws between autonomy considerations and best interest judgements is unduly rigid.

Why are we required to respect patients’ autonomy? We can infer from Beauchamp and Childress’s discussions that, in keeping with the standard way of thinking about the value of autonomy, it is morally imperative to respect patients’ autonomy because doing so is *inherently* of value. I do not disagree with this. What I take issue with is the idea that this is the only way in which respecting patients’ autonomy might be of value.

Respecting patients’ autonomy might also be *instrumentally* valuable. That is, the exercise of autonomy by patients in healthcare contexts might be of value because, as a consequence, their welfare will be promoted through “preference satisfaction” (Sandman and Munthe 2009, 302). This is consistent with previous empirical research that shows that being able to choose and having a level of control increases the physical and psychological well-being of residents in NHs (Schulz 1976).

For a trivial example, consider the case of a resident with dementia in a NH who has been asked by the staff about what she wants to eat for lunch. Here, seeking the resident’s preference for what she wants to eat is inherently valuable because we value exercising autonomy in and of itself. But satisfying the resident’s preference seems to be also instrumentally valuable: we expect that satisfying her preference for what she wants to eat promotes her welfare.

Accepting the above suggestion, in the context of care for PWD with diminished capacity for autonomous decision-making, weakens the presumed dichotomy between autonomy considerations and best interest considerations. Concern for the autonomy of PWD, in the sense explained above, promotes their welfare. It follows, then, that in order to contribute to the welfare of PWD, we need to take their current preferences seriously. This implies that we might not be constantly faced with a stark choice whether to give more priority to autonomy considerations or to best interest considerations.

What do all the foregoing discussions suggest in terms of finding a solution to the third problem of Beauchamp and Childress’s account?

The foregoing discussions suggest that the problem, in many cases, arises from not adequately taking account of current preferences of PWD, considering precedent autonomy as prescriptive, and drawing a rigid distinction between autonomy considerations and best interest judgements. If we make all the three modifications discussed above, our task in acting upon autonomy considerations regarding PWD with diminished capacity for autonomous decision-making seems to be easier than initially described by Beauchamp and Childress. Our task is not mainly to decide whether we should prioritize precedent autonomy or best interest judgements, which proved to be challenging. Our task entails taking individuals’ extant autonomy seriously insofar as it allows us to better interpret the requirements of their precedent autonomy because this, in turn, to some extent, promotes their welfare.

So far, my discussions have been related to PWD with diminished capacity for autonomous decision-making. Now one might raise the question as to whether these discussions also help us to resolve the conflict between precedent autonomy and best interest considerations for PWD in NHs whose decisions can be properly categorized as non-autonomous. As I argued above, these residents do not present us with any autonomy considerations other than the mandates of their precedent autonomy. As such, we cannot appeal to their extant autonomy to bridge the dichotomy between precedent autonomy and best interest considerations.

I concede that, regarding those individuals, we have a more difficult task to assess whether we should prioritize precedent autonomy or best interest judgements in cases of conflict between these considerations. But it seems to me that adopting a hermeneutic approach to the mandates of their precedent autonomy still offers some help in tackling this task. The hermeneutic approach, for example, does not construe the content of advance directives as rules to be followed, an approach which might increase the chance that the mandates of individuals’ precedent autonomy come into conflict with best interest judgements.

Before concluding my discussions in this paper, it seems helpful to compare the revised version of Beauchamp and Childress’s account of autonomous decision-making with the original version. Table 1 lists the main features of the original version of Beauchamp and Childress account vis-à-vis the main features of the revised account.

**Table 1:** A comparison between the revised version of Beauchamp and Childress’s account of autonomous decision-making with the original version in the context of decision-making by people with dementia in nursing homes

Main features of the original version of Beauchamp and Childress’s account	Main features of the revised account
• Intentionality as a binary criterion of autonomous decision-making	• Intentionality as a continuum criterion of autonomous decision-making
• Consent as one-off authorization	• Process consent
• A prescriptive approach to the mandates of precedent autonomy • General primacy of the mandates of precedent autonomy over contemporaneous expressions of autonomy and best interest considerations	• A hermeneutic approach to the mandates of precedent autonomy • Accounting for both precedent and extant autonomy • Interpreting mandates of precedent autonomy by reference to contemporaneous expressions of autonomy • Drawing a less rigid distinction between autonomy considerations and best interest considerations

## Conclusion

In this paper, I have argued that a revised version of Beauchamp and Childress’s account of autonomous decision-making fares better than the original version in capturing relevant autonomy and consent considerations to care for PWD in NHs. Below, I provide a brief summary of these autonomy-related considerations regarding each of the three categories of autonomous decision-making.

First, I claimed that we need to consider implementing a model of process consent for PWD whose decisions can be regarded as autonomous. This model ensures that there are regular consensual engagements with the patients vis-à-vis changes to their treatment plans.

Second, for those residents with diminished capacity for autonomous decision-making, I defended a two-component type of autonomy consideration, which accounts for both precedent and extant autonomy of the individuals. Along with this, I also made two additional claims: (1) it is more useful to adopt a hermeneutic approach to the mandates of precedent autonomy, and (2) that taking seriously the individuals’ extant autonomy, to some extent, promotes their welfare. I argued that these could minimize the possibility that autonomy considerations come into conflict with best interest considerations.

And third, for those residents with dementia whose decisions are considered non-autonomous, I agreed with Beauchamp and Childress that the mandates of their precedent autonomy are the only autonomy-related considerations. Nonetheless, I claimed that adopting a hermeneutic approach to precedent autonomy still might decrease the chance that the mandates of their precedent autonomy come into conflict with best interest considerations.

## Footnotes

1 Here and thereafter, by “decisions,” I primarily mean decisions about treatment and medical care such as whether or not to receive psychotropic medications in NHs.

2 It is worth noting that Beauchamp and Childress do not clarify in detail the distinction between doubtfully autonomous and non-autonomous decisions.

3 It is worth noting that Beauchamp and Childress ground the ethical justification for obtaining informed consent on protecting autonomous choice in healthcare contexts. This description is in keeping with the prevailing thinking about informed consent in biomedical ethics. There are, however, scholars who do not share this dominant view. For instance, O’Neill (2003) thinks that the moral justification for obtaining informed consent in healthcare contexts is to ensure that patients are not coerced or deceived. Another divergent view from the dominant thinking is the one supported by Taylor (2004) who argues that the moral justification for informed consent is the concern for patients’ well-being in healthcare contexts.

4 Here I assume that we need to respect individuals’ precedent autonomy once they lose some or all of their capacity for autonomous decision-making. This assumption, however, has drawn some criticisms. For instance, it might be argued that, particularly when there are significant psychological changes, individuals in their current status are in some sense different from their past selves. The implication, then, is that mandates of precedent autonomy such as those included in individuals’ advance directives lose their moral authority. For an insightful discussion of this objection and its rebuttal, see DeGrazia (1999).

5 For another account of why we should take seriously extant autonomy of people with (mild to moderate) dementia, see Jaworska (1999).

6 Note that this does not mean the practice of covert administration of the psychotropic medications may not be, all things considered, ethically justifiable. All I argue here is that this practice entails not paying due respect to patients’ extant autonomy.
